# Spatial distribution of tree species in evergreen-deciduous broadleaf karst forests in southwest China

**DOI:** 10.1038/s41598-017-15789-5

**Published:** 2017-11-15

**Authors:** Hu Du, Fang Hu, Fuping Zeng, Kelin Wang, Wanxia Peng, Hao Zhang, Zhaoxia Zeng, Fang Zhang, Tongqing Song

**Affiliations:** 1 Key Laboratory of Agro-ecological Processes in Subtropical Region, Institute of Subtropical Agriculture, Chinese Academy of Sciences, Changsha, 410125 Hunan China; 2 Huanjiang Observation and Research Station for Karst Ecosystems, Institute of Subtropical Agriculture, Chinese Academy of Sciences, Huanjiang, 547100 Guangxi China

## Abstract

Understanding the spatial distribution of tree species in subtropical evergreen-deciduous broadleaf karst forest is fundamental to studying species coexistence and karst species diversity. Here, complete spatial randomness and heterogeneous Poisson process models were used to analyze the spatial distribution patterns of 146 species with at least one individual per ha in a 25-ha plot in southwest China. We used canonical correspondence analysis (CCA) and the torus-translation test (TTT) to explain the distributions of observed species. Our results show that an aggregated distribution was the dominant pattern in Mulun karst forests; the percentage and intensity of aggregated decreased with increasing spatial scale, abundance, mean diameter at breast height (DBH), and maximum DBH. Rare species were more aggregated than intermediately abundant and abundant species. However, functional traits (e.g., growth form and phenological guild) had no significant effects on the distributions of species. The CCA revealed that the four analyzed topographic variables (elevation, slope, aspect, and convexity) had significant influences on species distributions. The TTT showed that not all species have habitat preferences and that 68.5% (100 out of 146 species) show a strongly positive or negative association with at least one habitat. Most species were inclined to grow on slopes and hilltops.

## Introduction

The spatial distributions of tree species in forests and their influencing mechanisms have always been a hot topic in research, as such information can help us to understand the potential ecological processes that control species coexistence and community structure^1,2^. Tree species can be distributed in an aggregated, a random, or a regular pattern, with aggregated distributions being widespread in natural forests, particularly tropical forsts^1,3–5^. Over the past few decades, a number of mechanisms have been found to contribute to species aggregation in forests, including niche segregation^6^, habitat heterogeneity^7^, dispersal limitation^8^, intra- and inter-species competition^9^, and negative density dependence^10^.

Habitat heterogeneity and dispersal limitation are among the most influential ecological processes. Recently, numerous studies have demonstrated that many species are limited to a range of habitats^11^. Worldwide, research in forest plots has shown that habitat factors and microhabitat heterogeneity (e.g., topographical factors, rock outcrops, and soil heterogeneity) play vital roles in the spatial patterns of tree species^7,12,13^, and some studies have shown that many species have a significantly positive or negative relationship with slope, elevation, or aspect^7,14,15^. Furthermore, spatial distribution can also be affected by differences in functional traits (e.g., growth form, shade tolerance, and seed dispersal limitation) and ecological strategies^16–19^. For example, the mode of seed dispersal affects the spatial distribution of trees, with species dispersed by animals being less aggregated than those dispersed by wind or gravity^3,4^. Moreover, species’ attributes also influence spatial patterns. For example, trees with larger trunk diameters are less aggregated^3,4^, indicating self-thinning. Therefore, the distribution patterns of tree species are controlled by multiple factors in the forest ecosystem.

The current knowledge regarding spatial distributions and their underlying mechanisms in species-rich communities has mostly been derived from tropical^20–22^ and subtropical^4,11^ forests. Similar studies on the species spatial distributions of species and species-habitat associations in other specific forest types (e.g., karst forest) are insufficient due to a lack of large forest plots. The karst landscape is mainly found in Eastern Europe, the European Mediterranean, North America, and southwest China, the latter of which has the largest and widest karst area in the world^23^. Mixed evergreen-deciduous broadleaf forest is unique and representative of the karst landscape in southwest China. This forest type has a complicated community structure, rich biodiversity, and high habitat heterogeneity^23–26^, making it an ideal community in which to study spatial patterns. However, our current understanding of spatial patterns in karst forests has mainly been derived from studies conducted at small scales ( ≤ 1 ha). Consequently, the community-wide patterns of the spatial distributions of species and species-habitat associations and the underlying mechanisms of species coexistence are not well understood in karst forests^5^.

The last undisturbed remnants of karst forest in China (and possibly around the world) are mainly located in Mulun National Natural Reserve and Maolan National Natural Reserve in Guangxi and Guizhou province. Mixed evergreen-deciduous broadleaf forest is a typical subtropical forest in the karst region of southwest China. According to the census measure for the Barro Colorado Island (BCI) plot in Panama, a 25 ha dynamic forest plot was established and surveyed in Mulun in 2014. The Mulun plot is a component of the Chinese Forest Biodiversity Monitoring Network (CForBio) and is also the largest forest plot in the karst region. The main objectives of this study were to (1) analyze the spatial distribution patterns of tree species at different scales in the 25 ha plot using the complete spatial randomness (CSR) null model and homogeneous Poisson process (HPP) null model, (2) examine whether species attributes (e.g., species abundance, diameter at breast height (DBH) class, growth form, etc.) are related to their spatial distribution patterns, and (3) examine species-habitat associations in different habitat types. This study will contribute to our understanding of species coexistence and diversity in subtropical karst forests.

## Results

**Figure 1:**
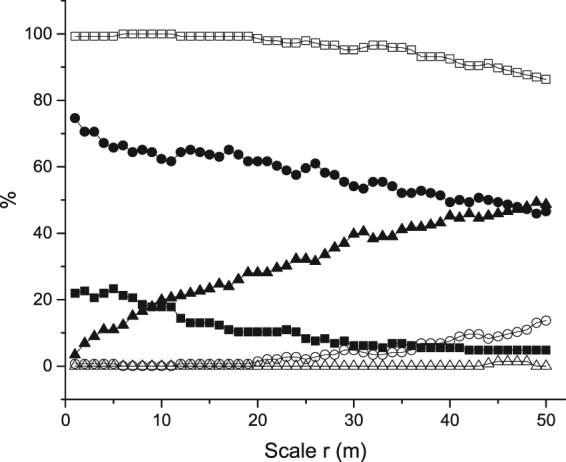
Proportions of species showing significant aggregated (square), random (circle), and regular (triangles) distributions at scales of from 0 to 50 m based on the complete spatial randomness (CSR) (open symbols) and heterogeneous Poisson process (HPP) (solid symbols) models for the 25 ha Mulun plot.

**Figure 2:**
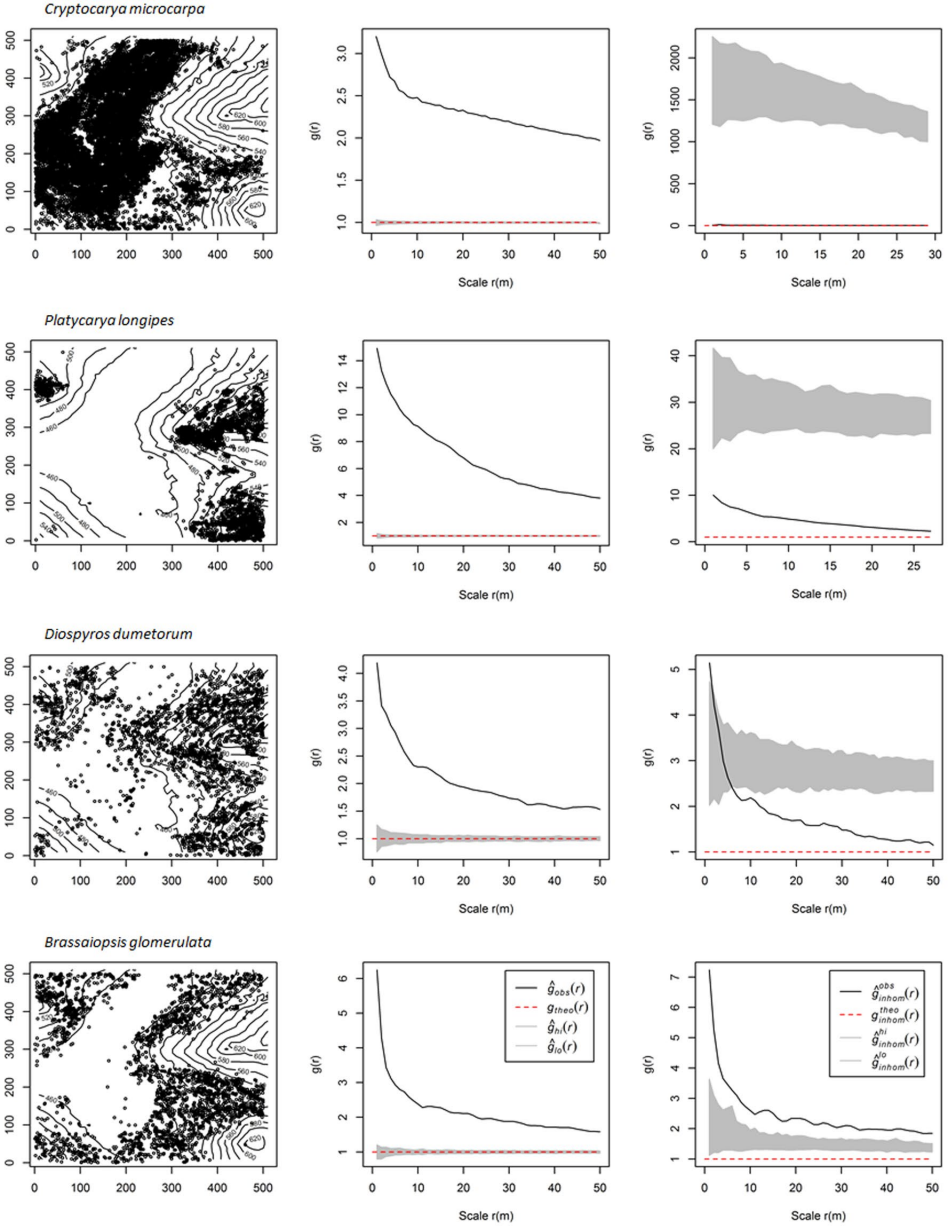
Four examples of species distributions in the Mulun plot. Left panels show corresponding distribution patterns. Middle and right panels show the relationship between the univariate pair-correlation function (*g*(*r*)) and scale for the four species. The lines represent *g*(*r*); the gray areas indicate the simulation envelopes generated from 999 Monte Carlo simulations under the null hypothesis of complete spatial randomness (CSR, the middle panels) and heterogeneous Poisson process (HPP, the right panels). The figures were created using R 3.3.2 software^31^ (https://www.r-project.org/).

All species were aggregated at small scales, some even up to 50 m, and no species showed regular patterns under the CSR null model, with two exceptions: *Loropetalum chinense*, which showed a regular pattern at from 45 to 48 m, and *Clerodendrum japonicum*, which showed a regular pattern at from 44 to 48 m (See Appendix Table S1 for details and Fig. 1). The percentage of aggregated species decreased with an increase in the spatial scale: 146 species (100%) were aggregated at a scale of 6 to 11 m, 145 species (99.3%) were aggregated at 0 to 19 m, 139 species (95.2%) were aggregated at 36 m, and 126 species (86.3%) were aggregated at 50 m (Fig. 1). In contrast, the percentage of species occurring randomly increased at scales of 12 to 50 m. Additionally, the species showed different aggregation intensities, with the four dominant species being suited to different habitats (Fig. 2). *Cryptocarya microcarpa* was aggregated in depressions, *Platycarya longipes* on hilltops, and *Brassaiopsis glomerulata* on slopes.

To obtain a rough estimate of the magnitude of the large-scale effects of habitat heterogeneity on local tree density, all species distributions were examined and contrasted with the HPP null model. More than 45.9% of the species showed random patterns at 50 m, fewer than 23.5% of the species showed aggregated patterns at 50 m, and five species (3.4%) showed significant regularity at all scales (0 to 50 m). The percentages of aggregated and randomly occurring species decreased with increasing scale, whereas the percentage of regularly occurring species increased with scale (Fig. 1).

**Figure 3:**
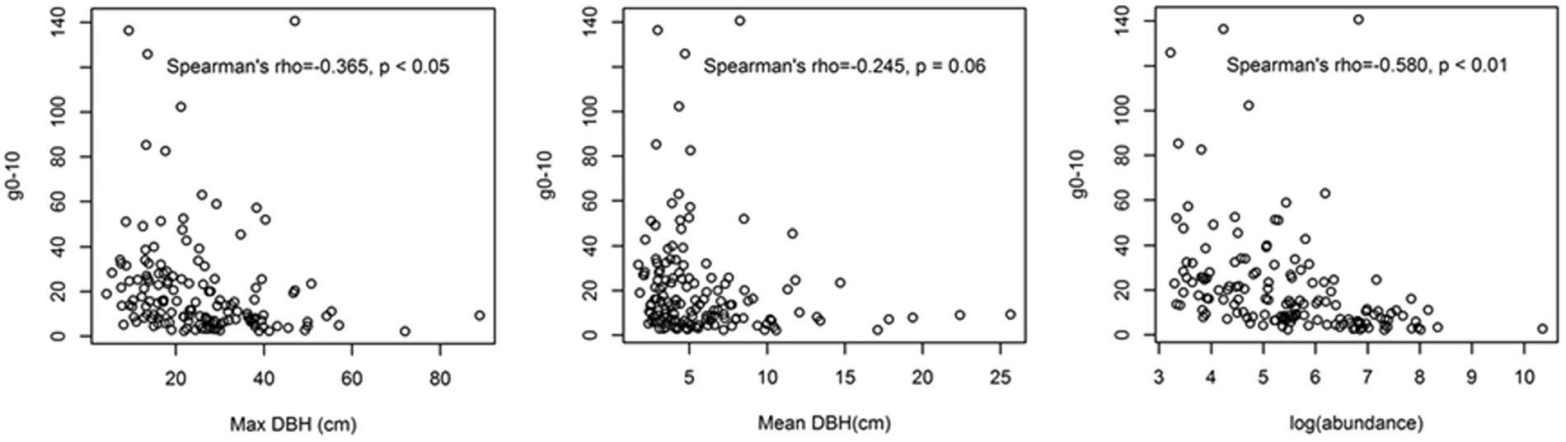
Relationships between the aggregation index (g0-10) and maximum DBH, mean DBH, and abundance of species with an abundance ≥25 in the Mulun plot.

Based on the CSR null model, aggregation intensity showed a negative relationship with species abundance, mean DBH, and maximum DBH, i.e., the aggregation intensity declined with these factors (Fig. 3). There was a clear trend that aggregation intensity declined with species abundance (Spearman’s rho = −0.580, p < 0.01): rare species (<4 individuals/ha, n = 43, mean = 33.16, SE = 4.3) were more aggregated than intermediate (4–40 individuals/ha, n = 78, mean = 18.5, SE = 2.48) and abundant species ( ≥ 40 individuals/ha, n = 24, mean = 7.6, SE = 1.06). The results of the Kruskal-Wallis test show that the aggregation intensity of rare species was significantly different from that of intermediate and abundant species (Kruskal-Wallis χ^2^ = 39.913, p < 0.001).

The average aggregation intensity of (*g*0-10) of canopy species (n = 26, mean = 8.07, SE = 5.47) was lower than that of midstory species (n = 64, mean = 20.42, SE = 2.97) and understory species (n = 55, mean = 23.23, SE = 3.00) for the CSR null model. However, there were no significant differences among the three growth forms (ANOVA; F = 0.473, p = 0.624).

The mean g0-10 of evergreen species (n = 95, mean = 24.6, SE = 2.7) was higher than that of deciduous species (n = 50, mean = 14.2, SE = 2.1) for the CSR null model. However, the aggregation intensities of deciduous and evergreen species were not significantly different (W = 3,145, p = 0.999) based on the CSR null model. The average g0-10 of endemic species (n = 18, mean = 22.1, SE = 4.2) was higher than that of non-endemic species (n = 127, mean = 20.8, SE = 2.2) for the CSR null model. However, the aggregation intensities of endemic species and non-endemic species were not significantly different (t = 0.266, p = 0.792) based on the CSR null model.

**Figure 4:**
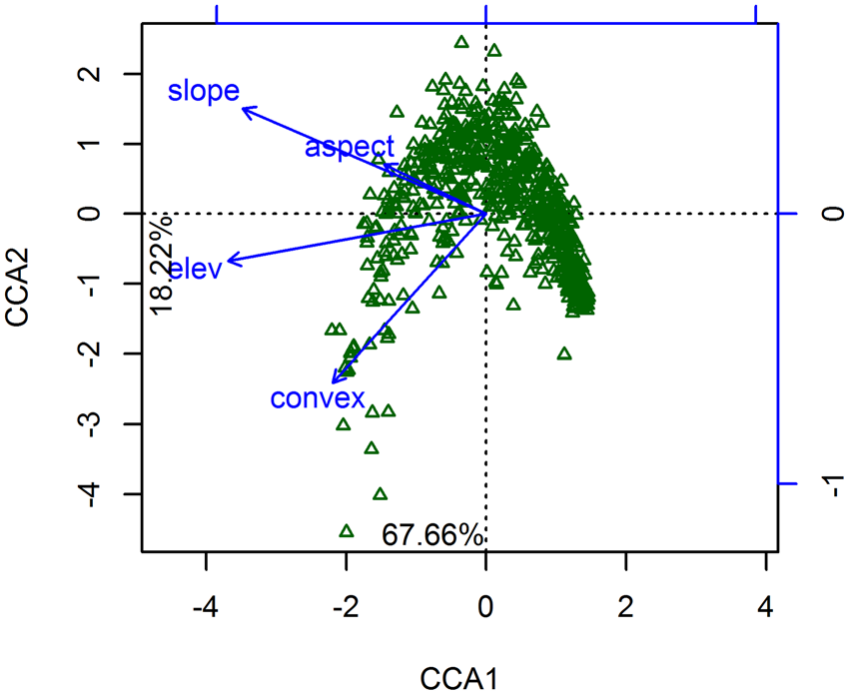
Canonical correspondence analysis (CCA) biplot of the 146 species and topographic factors.

Our results show that four selected topographic factors significantly affect the species distributions, with the four factors explaining 17.29% of the variation in tree species distributions (Table 1). The spatial distributions of the tree species were significantly negatively correlated to these topographic factors on the first axis and significantly positively correlated to slope and aspect but not elevation and convexity on the second axis (Fig. 4).

**Table 1:** Permutation test for the topographic factors explaining the distributions of woody plants in the Mulun plot.

Topographic factor	CCA1	CCA2	*R* ^2^	*Pr* (>r)
Elevation	−0.987	−0.160	0.878	0.001***
Slope	−0.983	0.180	0.788	0.001***
Aspect	−0.978	0.205	0.141	0.001***
Convexity	−0.848	−0.531	0.538	0.001***

***p < 0.001; *P* is the result of a permutation test run 1000 times; CCA1 and CCA2 are the results for the first and second axes of the ordination; *R* ^2^ is the determination coefficient for the topographic factors; *Pr* represents the significance from the correlation test.

**Figure 5:**
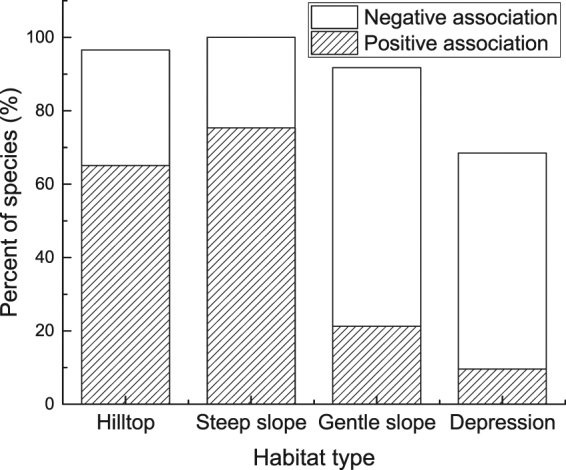
Torus-translation tests for habitat association in the Mulun plot.

Different species were suited to different habitat types (Fig. 5). Based on the torus-translation tests (TTT), 95 (65.06%), 110 (75.34%), 31 (21.23%), and 14 (9.59%) out of 146 species were significantly positively associated with hilltops, steep slopes, gentle slopes, and depressions, respectively, while 46 (31.51%), 36 (24.66%), 103 (70.55%), and 86 (58.90%) species were significantly negatively associated with these habitat types.

## Discussion

An aggregated distribution was the main spatial pattern in natural forests, while only a few species were randomly distributed, and few species showed a regular distribution^2–4,27–30^. Aggregation was also the main distribution pattern among dominant species in this study. Among 146 species (with at least one individual/ha), 145 (approximately 99.3%) were aggregated at scales of from 0 to 19 m. Compared with other studies, the intensity of aggregation in the Mulun plot was higher than that in temperate forests and similar to that in subtropical and tropical forests. For example, approximately 99.2% of species were aggregated at scales of from 0 to 10 m in six tropical forest plots^3^, 99.3% and 98% of species were aggregated in two subtropical forests^4,28^, and 90.5% of species were aggregated in a temperate forest^31^. The percentage of aggregated species and the intensity of aggregation species declined with an increase in spatial scale. Similar results were also found in subtropical^28^ and temperate forests^27^. These phenomena fully confirm the existence of scale separation.

To test the effects of habitat heterogeneity, we used HPP as the null model. Based on the HPP model, only seven of the 146 species were aggregated at all scales from 0 to 50 m, while five species were regularly distributed at these scales. After eliminating topographic factors (elevation, slop, convexity, and aspect), fewer than 23.5% species were aggregated, and random was the most common distribution pattern. In comparison to other studies, the percentage of species showing an aggregated pattern at scales of from 0 to 10 m (17.81%) in this study was lower than that observed in tropical karst seasonal rainforest (28.85%)^32^ and far lower than that recorded in a subtropical mixed evergreen-deciduous broadleaf forest in a non-karst region (58%)^28^. The considerable habitat heterogeneity in karst regions is perhaps the main factor driving species distributions. Therefore, habitat heterogeneity is an important mechanism regulating species aggregation patterns based on the results of the CSR and HPP null models.

Numerous factors play a vital role in determining the spatial patterns of trees in a plant community. Such spatial patterns could arise from many biotic and abiotic processes, such as regeneration, habitat heterogeneity, dispersal limitation, and disturbance^3,33,34^. Our study showed that species abundance, DBH, growth form, and phenological guild were important factors affecting the spatial patterns of trees in a subtropical karst forest. We analyzed the relationships between species’ attributes (abundance, mean DBH, and maximum DBH) and aggregation intensity for the 145 species aggregated at scales of from 0 to 10 m based on the CSR null model. This analysis indicated that rare species were more aggregated than intermediate and abundant species, a result that is consistent with those from other studies^2–5,27,28^. However, not all species showed a similar trend: the rare species *Toona sinensis* and *Choerospondias axillaris*, with 74 and 47 individuals, respectively, had relatively low *g*0-10 values of 7.12 and 7.95, respectively, while the abundant species *Tirpitzia ovoidea* had a relatively high *g*0-10 value of 24.61 with 1,299 individuals. One of the most important reasons for such patterns is that the spatial distributions of some species can arise from strong habitat preferences^23^. Thus, species distribution patterns are species-specific and may occur as a result of a variety of biological and ecological processes, such as stochastic recruitment, clonality, competition, patchy habitat heterogeneity, disturbance, and other stochastic events^32,35,36^. Our results show that the aggregation intensity *g*0-10 decreased with increasing DBH due to self-thinning or density-dependent mortality^30^, which is consistent with previous studies^2,28^. Water and soil were the limiting resources in karst forest^26^, and adjacent trees are likely to compete with one another for these limiting resources. Furthermore, larger trees competitively inhibit conspecifics over a larger area of influence than smaller trees^5^. Thus, small-sized trees were more clumped than larger trees.

Tree height is important for seed dispersal because the distance traveled by seeds is a function of the height of reproductive structures. The higher the stature, the longer and more variable the dispersal distance^37^. Previous studies have shown that understory species usually have more aggregated patterns than canopy species due to inefficient seed dispersal^27^. We also found that understory species were the most aggregated species in the Mulun plot, followed by midstory and canopy species, but there were no significant differences among these growth forms. Furthermore, evergreen species were more aggregated than deciduous species in our study, which is consistent with other studies^28,32^, but the distributions of deciduous and evergreen species were not significantly different. This discrepancy between this and other studies might be related to high habitat heterogeneity and the unique geology of the karst region, which may form strong associations between species habitats and their distributions.

The canonical correspondence analysis (CCA) results show that the spatial distributions of species in Mulun plot can be significantly explained by four topographic factors (elevation, slop, convexity, and aspect). The combination of these factors explained 17.29% of the species distributions; however, edaphic or other environmental factors can also play an important role in governing species distributions^7^. Furthermore, the TTT results show that not all species have habitat preferences, which is consistent with other studies^2,7,14,38^. Approximately 68.5% (100 out of 146) species showed strongly positive or negative associations with at least one habitat, demonstrating that habitat heterogeneity plays a vital role in regulating the spatial distributions of species in subtropical karst forest.

Our results show that over 134 species in the Mulun plot have a distinctly positive or negative correlation with hilltops (141 species), steep slopes (146 species), and gentle slope (134 species), while only 100 species were significantly positively or negatively associated with depressions. Moreover, we also found that greater numbers of species were positively associated with hilltops (67.4%) and steep slopes (75.3%) and negatively associated with gentle slopes (76.8%) and depressions (86.0%). These results indicate that most species were inclined to grow on slopes and hilltops; similar results have been found in tropical karst seasonal rain forest^39^ due to the high light and temperatures at higher elevations. The ecological conditions of different habitat types, along with topographic factors of the terrain, may influence species distribution patterns through the redistribution of light, temperature, soil moisture, and nutrients^2^. At our study site, complex terrain conditions may lead to habitat specialization, and each species has its preferred habitat. For example, *Platycarya longipes* establishes more successfully on hilltops. Therefore, habitat partitioning caused by topography may be a major mechanism allowing the coexistence of trees in the Mulun plot. Our results provide evidence that species attributes and habitat heterogeneity jointly contribute to the regulation of their spatial distributions in subtropical evergreen deciduous broadleaf karst forest in southwest China.

## Methods

### Study site

The study was conducted at the Mulun National Reserve (Mulun Reserve) (107°54′01″-108°05′51″E, 25°07′01″-25°12′22″N), in Huanjiang county, northwestern China’ Guangxi Province. Mulun Reserve was established in 1991 to protect the subtropical mixed evergreen–deciduous broadleaf forest ecosystem that has developed on the limestone substrate. In this 10, 800 ha reserve, the topography is characterized by steep hills separated by lowland depressions with numerous potholes and caves and extensive underground streams. This area has a subtropical climate with an average annual temperature of 19.38 °C and average annual precipitation of 1,500 mm, occurring mainly from April to August. The mean annual frost-free period lasts 310 days, and the mean annual relative humidity is 79%.

**Figure 6:**
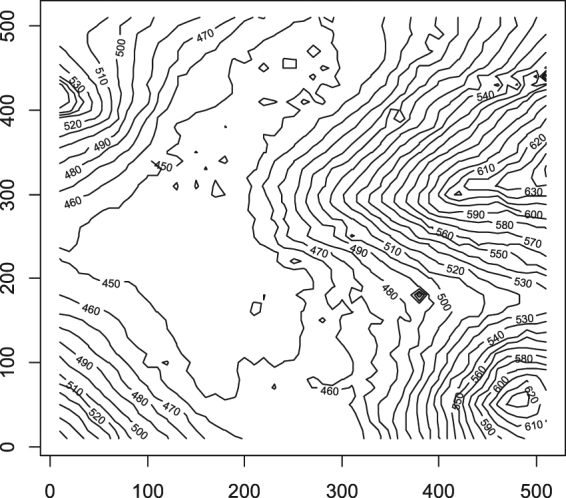
Contour map of the 25 ha Mulun forest plot. The numbers in the map represent elevation (m). The map was created using R3.3.2 software^31^ (https://www.r-project.org/).

A 25 ha (500 m × 500 m) plot (25°8′N, 108°0′E) was established in Mulun Reserve in April 2014, with the first census completed in December 2014 following the standard field protocol of the CTFS (Center for Tropic Forest Sciences, http://www.ctfs.si.edu). The plot is characterized by rugged terrain (Fig. 6): the altitude varies from 442.6 m to 651.4 m, and the slope ranges from 0.12° to 66.97°, with a mean of 31.4°. The average rock exposure ratio is greater than 60%.

### Data collection

The plot was divided into a grid composed of 625 20 × 20 m cells, and all woody stems with a DBH ≥ 1 cm were identified, tagged, measured, and mapped following standard field procedures^40^. According to the first census, the plot had 144,552 individuals belonging to 51 families, 127 genera, and 228 species. Important species included *Cryptocarya microcarpa*, *Itoa orientalis*, *Platycarya longipes*, and *Lindera communis*. Species abundances varied to a great extent, from 1 to 31,486 individuals. To obtain a sufficient sample size for point pattern analyses, we chose 146 common species with no fewer than 25 individuals^28^.

### Data analyses

#### Spatial point pattern analysis

Spatial point pattern analyses have been widely used to analyze the spatial patterns of tree distribution^2^. In this paper, we used *g*(*r*) to study the spatial distributions of the study species on a 0 to 50 m scale^28,41^ and mean *g*0-10 as a measure of the mean conspecific aggregation density within 10 m of a tree^3^. The *g* function is derived from Ripley’s *K* function, which is the probability density of the *K* function. It is a non-cumulative distribution function and presents the expected density of trees in a circular ring of a given distance *r* around a focal tree, divided by the intensity of the pattern^37^.

We used CSR and HPP as null models. The former null model is widely used for univariate point patterns; it assumes no interactions between points and indicates that trees can occur at any position without the influence of biological processes^42^. The latter is used to investigate the significant second-order effects of the spatial distribution of tree species. It can predict the probability of occurrence of a tree of a given species in space, as a function of environmental covariates. In this study, four topographic variables represented the environmental covariates in each 10 × 10 m quadrat: elevation, slope, aspect, and convexity^28^. Elevation was the mean elevation of the corners of the quadrat; slope was the true value obtained through measurement at the central points in each subplot; aspect was the compass direction to which the slope faced; and convexity was the difference between the mean elevation of a focal quadrat and the average elevation of the eight surrounding quadrats^7,38^. The significance of departure from a null model was tested using 999 random Monte Carlo simulations in the “spatstat” package^43^ in R. If the observed value fell outside the 5th and 95th quartiles, the null model could not be rejected and the species is considered to be aggregated.

We used the mean *g*0-10 to compare the distribution patterns of different growth forms, phenological guilds (evergreen species and deciduous species), and endemic and non-endemic species and then used the Kruskal-Wallis or Wilcoxon rank sum test to test the significance. The observed species were divided into three growth forms according to their mean DBH^28^: understory species (<4 cm DBH; 55 species), midstory species (≥4 and <8 cm DBH; 64 species), and canopy species (≥8 cm DBH; 26 species). Additionally, we divided the abundance into three levels: abundant (with abundance ≥ 40 individuals/ha), intermediate (4–40 individuals/ha), and rare (<4 individuals/ha).

#### Analyses of topographic factors and habitat preference

**Figure 7:**
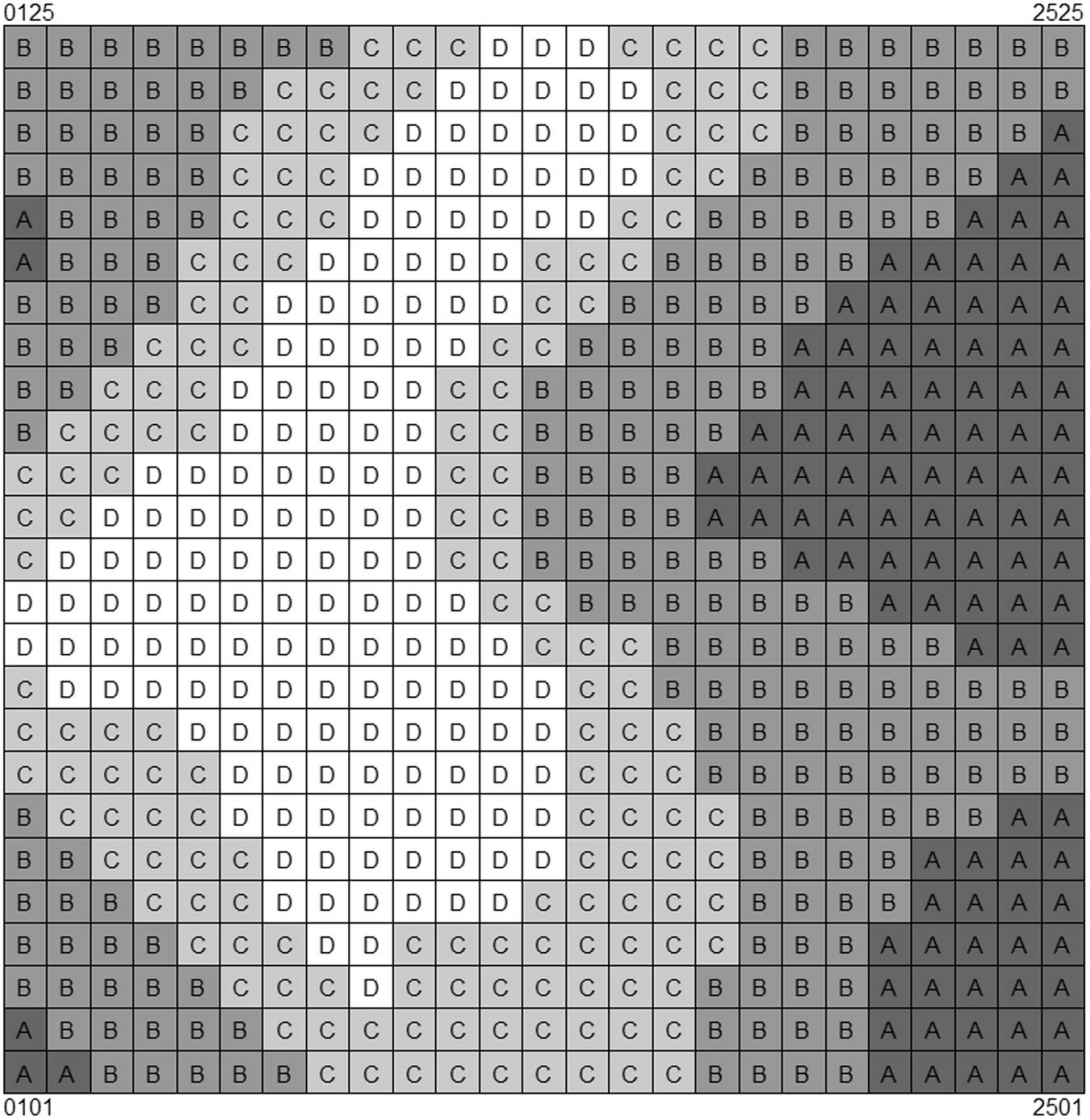
Grid map of the 25 ha plot based on 625 20 m × 20 m cells. (**A**) hilltop, (**B**) steep slope, (**C**) gentle slope, (**D**) depression).

The elevation, slope, aspect, and convexity of each 20 × 20 m grid were selected as topographic variables for use in a species-habitat association analysis^2^. We used a multivariate regression tree technique to divide the 625 cells into four habitat types: hilltop, steep slope, gentle slope, and depression (Fig. 7) using package “rpart” in R^39^.

We performed a CCA to analyze the relationship between topography and the spatial distribution of woody plants and employed a Monte Carlo permutation test to evaluate the significance of these relationships using the “vegan” package in R. Each topographic variable was tested at the 5% significance level using 1,000 random permutations. Due to the autocorrelation among species distributions, we used the TTT method to analyze the relationship between species and habitat^7^. Detailed descriptions of the TTT can be found in Harms *et al*.^7^.

All analyses were performed using R3.3.2^31^ (R Development Core Team) and Microsoft Excel 2013.

## Electronic supplementary material

Supplementary Information
