# Supplementary material for: Spatial distribution of tree species in evergreen-deciduous broadleaf karst forests in southwest China
Source: Sci Rep. 2017 Nov 15;7:15664. doi: 10.1038/s41598-017-15789-5 (PMC5688135; doi:10.1038/s41598-017-15789-5)
Supplement: Supplementary file 1 — Supplementary Information [file 41598_2017_15789_MOESM1_ESM.doc]

**Spatial distribution of tree species in evergreen-deciduous broadleaf karst forests in southwest China**

Hu Du^1,2^, Fang Hu^1,2^, Fuping Zeng^1,2^, Kelin Wang^1,2^, Wanxia Peng^1,2^, Hao Zhang^1,2^, Zhaoxia Zeng^1,2^, Fang Zhang^1,2^ & Tongqing Song^1,2^

^1^Key Laboratory of Agro-ecological Processes in Subtropical Region, Institute of Subtropical Agriculture, Chinese Academy of Sciences, Changsha 410125, Hunan, China. ^2^Huanjiang Observation and Research Station for Karst Ecosystems, Institute of Subtropical Agriculture, Chinese Academy of Sciences, Huanjiang 547100, Guangxi, China.

Correspondence and requests for materials should be addressed to T.Q. (email: [songtongq@isa.ac.cn](mailto:songtongq@isa.ac.cn))

Table S1 Analyses of complete spatial randomness (CSR), heterogeneous Poisson null model (HPP), and spatial patterns for 146 species in the 25 ha Mulun plot

|  | SCR | | |  | HPP | | |
| --- | --- | --- | --- | --- | --- | --- | --- |
| Species name | AG | RA | RE |  | AG | RA | RE |
| *Acer sycopseoides* | 0-50 |  |  |  |  |  | 0-50 |
| *Acer wangchii* | 0-50 |  |  |  | 0-35 | 36-50 |  |
| *Acer yinkunii* | 0-50 |  |  |  |  | 0-50 |  |
| *Actinodaphne cupularis* | 0-50 |  |  |  |  | 0-50 |  |
| *Adenanthera pavonina* | 0-50 |  |  |  |  | 0-50 |  |
| *Alangium chinense* | 0-49 | 50 |  |  |  | 0-45 | 46-50 |
| *Albizia calcarea* | 0-50 |  |  |  |  | 0-50 |  |
| *Alchornea trewioides* | 0-50 |  |  |  | 0-2 | 3-31 | 32-50 |
| *Aralia chinensis* | 0-28 | 29-50 |  |  |  | 0-50 |  |
| *Ardisia thyrsiflora* | 0-50 |  |  |  |  | 0-28 | 29-50 |
| *Bauhinia brachycarpa* | 0-50 |  |  |  | 0-50 |  |  |
| *Beilschmiedia intermedia* | 0-50 |  |  |  |  | 0-50 |  |
| *Bennettiodendron leprosipes* | 0-50 |  |  |  |  | 0-50 |  |
| *Boehmeria dolichostachya* | 0-36 | 37-50 |  |  |  | 0-50 |  |
| *Boniodendron minus* | 0-50 |  |  |  |  | 0-1 | 2-50 |
| *Brassaiopsis glomerulata* | 0-50 |  |  |  | 0-50 |  |  |
| *Bridelia insulana* | 6-50 | 0-5 |  |  |  | 0-50 |  |
| *Bridelia retusa* | 0-50 |  |  |  | 0-7 | 8-50 |  |
| *Bridelia tomentosa* | 0-50 |  |  |  | 0-50 |  |  |
| *Buddleja officinalis* | 0-50 |  |  |  |  | 0-50 |  |
| *Callicarpa bodinieri* | 0-40 | 41-50 |  |  |  | 0-50 |  |
| *Callicarpa longifolia* | 0-50 |  |  |  | 0-36 | 37-50 |  |
| *Canthium dicoccum* | 0-50 |  |  |  | 0-1 | 2-50 |  |
| *Carpinus luochengensis* | 0-50 |  |  |  |  | 0-6 | 7-50 |
| *Carpinus pubescens* | 0-50 |  |  |  |  | 0-9 | 10-50 |
| *Carpinus turczaninowii* | 0-50 |  |  |  |  | 0-50 |  |
| *Celtis sinensis* | 0-50 |  |  |  |  | 0-7 | 8-50 |
| *Celtis timorensis* | 0-40 | 40-50 |  |  | 0-7 | 8-50 |  |
| *Choerospondias axillaria* | 0-44 | 45-50 |  |  |  | 0-50 |  |
| *Chukrasia tabularis* | 0-50 |  |  |  |  | 0-17 | 18-50 |
| *Cinnamomum austrosinense* | 0-33 | 34-50 |  |  | 0-5 | 6-50 |  |
| *Cinnamomum migao* | 0-50 |  |  |  |  | 0-9 | 10-50 |
| *Cinnamomum porrectum* | 0-50 |  |  |  |  | 0-50 |  |
| *Cinnamomum saxatile* | 0-50 |  |  |  |  | 0-10 | 11-50 |
| *Cinnamomum wilsonii* | 0-50 |  |  |  | 0-13 | 14-50 |  |
| *Cipadessa cinerascens* | 0-50 |  |  |  | 0-11 | 12-50 |  |
| *Cladrastis platycarpa* | 0-50 |  |  |  | 0-50 |  |  |
| *Clausena dunniana* | 0-50 |  |  |  |  |  | 0-50 |
| *Cleidion bracteosum* | 0-50 |  |  |  | 0-30 | 31-50 |  |
| *Clerodendrum japonicum* | 0-11 | 12-45 | 46-50 |  |  | 0-47 | 48-50 |
| *Cordia dichotoma* | 0-50 |  |  |  |  | 0-50 |  |
| *Crateva unilocalaris* | 0-28 | 29-50 |  |  |  | 0-50 |  |
| *Croton tiglium* | 0-50 |  |  |  |  | 0-2 | 3-50 |
| *Cryptocarya microcarpa* | 0-50 |  |  |  |  |  | 0-50 |
| *Cyclobalanopsis glauca* | 0-50 |  |  |  | 0-12 | 13-26 | 27-50 |
| *Daphniphyllum longistylum* | 0-50 |  |  |  | 0-10 | 11-50 |  |
| *Decaspermum gracilentum* | 0-50 |  |  |  |  | 0-17 | 18-50 |
| *Diospyros dumetorum* | 0-50 |  |  |  | 0-1 | 2-6 | 7-50 |
| *Diospyros kaki* | 0-50 |  |  |  | 0-29 | 30-50 |  |
| *Distylium cuspidatum* | 0-50 |  |  |  |  | 0-3 | 4-50 |
| *Elaeocarpus japonicus* | 0-50 |  |  |  |  | 0-50 |  |
| *Engelhardtia roxburghiana* | 0-50 |  |  |  |  | 0-5 | 6-50 |
| *Eriobotrya japonica* | 0-44 | 45-50 |  |  |  | 0-39 | 40-50 |
| *Eriobotrya seguinii* | 0-50 |  |  |  |  | 0-12 | 13-50 |
| *Euonymus dielsianus* | 0-50 |  |  |  |  | 0-50 |  |
| *Eurycorymbus cavaleriei* | 0-50 |  |  |  |  | 0-7 | 8-50 |
| *Ficus erecta* | 0-50 |  |  |  |  | 0-3 | 4-50 |
| *Ficus oligodon* | 0-50 |  |  |  |  | 0-50 |  |
| *Ficus tinctoria* | 0-50 |  |  |  | 0-2 | 3-46 | 47-50 |
| *Ficus trichocarpa* | 0-50 |  |  |  |  | 0-50 |  |
| *Fraxinus insularis* | 0-50 |  |  |  |  | 0-41 | 42-50 |
| *Gleditsia fera* | 0-50 |  |  |  |  | 0-40 | 41-50 |
| *Gleditsia sinensis* | 0-43 | 44-50 |  |  | 0-8 | 9-50 |  |
| *Glochidion philippicum* | 0-50 |  |  |  |  | 0-50 |  |
| *Glycosmis parviflora* | 0-36 | 37-50 |  |  |  | 0-50 |  |
| *Gomphandra tetrandra* | 0-50 |  |  |  | 0-5 | 6-50 |  |
| *Handeliodendron bodinieri* | 0-30 | 31-50 |  |  | 0-19 | 20-50 |  |
| *Ilex bioritsensis* | 0-50 |  |  |  | 0-6 | 7-50 |  |
| *Ilex pentagona* | 0-20 | 21-50 |  |  |  | 0-50 |  |
| *Ilex stewardii* | 0-50 |  |  |  |  | 0-50 |  |
| *Itea coriacea* | 0-47 | 48-50 |  |  |  | 0-23 | 24-50 |
| *Itoa orientalis* | 0-50 |  |  |  |  |  | 0-50 |
| *Jatropha curcas* | 0-50 |  |  |  |  | 0-50 |  |
| *Koelreuteria bipinnata* | 0-50 |  |  |  | 0-17 | 18-50 |  |
| *Laurocerasus australis* | 0-50 |  |  |  | 0-8 | 9-29 | 30-50 |
| *Ligustrum japonicum* | 0-50 |  |  |  |  | 0-39 | 40-50 |
| *Ligustrum sinense* | 0-50 |  |  |  |  | 0-15 | 16-50 |
| *Lindera communis* | 0-50 |  |  |  | 0-24 | 25-50 |  |
| *Lindera megaphylla* | 0-50 |  |  |  |  | 0-50 |  |
| *Lindera pulcherrima* | 0-50 |  |  |  |  | 0-34 | 35-50 |
| *Liquidambar formosana* | 0-50 |  |  |  |  | 0-14 | 15-50 |
| *Lithocarpus harlandii* | 0-50 |  |  |  |  | 0-13 | 14-50 |
| *Litsea lancifolia* | 0-50 |  |  |  |  | 0-22 | 23-50 |
| *Loropetalum chinense* | 0-26 | 27-46 | 47-50 |  |  | 0-50 |  |
| *Luculia intermedia* | 0-50 |  |  |  |  | 0-3 | 4-50 |
| *Macaranga adenantha* | 0-50 |  |  |  | 0-4 | 5-37 | 38-50 |
| *Machilus pingii* | 0-50 |  |  |  |  | 0-27 | 28-50 |
| *Maesa japonica* | 0-50 |  |  |  |  | 0-36 | 37-50 |
| *Magnolia mulunica* | 0-50 |  |  |  | 0-25 | 26-50 |  |
| *Mallotus barbatus* | 0-45 | 46-50 |  |  |  | 0-50 |  |
| *Mallotus japonicus* | 0-50 |  |  |  |  | 0-50 |  |
| *Mallotus philippensis* | 0-50 |  |  |  | 0-3 | 4-39 | 40-50 |
| *Meliosma thorelii* | 0-50 |  |  |  |  | 0-10 | 11-50 |
| *Micromelum integerrimum* | 0-50 |  |  |  |  | 0-50 |  |
| *Miliusa chunii* | 0-50 |  |  |  | 0-50 |  |  |
| *Murraya paniculata* | 0-50 |  |  |  | 0-30 | 31-50 |  |
| *Myrsine semiserrata* | 0-50 |  |  |  | 0-4 | 5-41 | 42-50 |
| *Nandina domestica* | 0-50 |  |  |  | 0-7 | 8-18 | 19-50 |
| *Oreocnide frutescens* | 0-36 | 37-50 |  |  | 0-5 | 6-50 |  |
| *Oreocnide kwangsiensis* | 0-50 |  |  |  | 0-1 | 2-33 | 34-50 |
| *Pavetta hongkongensis* | 0-50 |  |  |  | 0-24 | 25-50 |  |
| *Phoebe calcarea* | 0-50 |  |  |  | 0-50 |  |  |
| *Phoebe crassipedicella* | 0-50 |  |  |  |  | 0-48 | 49-50 |
| *Phoebe neurantha* | 0-44 | 45-50 |  |  | 0-1 | 2-46 | 47-50 |
| *Photinia parvifolia* | 0-41 | 52-50 |  |  |  | 0-50 |  |
| *Photinia serrulata* | 0-50 |  |  |  |  | 0-50 |  |
| *Phyllanthodendron dunnianum* | 0-50 |  |  |  |  | 0-5 | 6-50 |
| *Picrasma quassioides* | 0-50 |  |  |  |  | 0-22 | 23-50 |
| *Pistacia* species No.1 | 0-50 |  |  |  |  | 0-6 | 7-50 |
| *Pistacia weinmannifolia* | 0-50 |  |  |  |  | 0-19 | 20-50 |
| *Pittosporum brevicalyx* | 0-50 |  |  |  |  | 0-15 | 16-50 |
| *Pittosporum kwangsiense* | 0-50 |  |  |  | 0-11 | 12-29 | 30-50 |
| *Pittosporum tonkinense* | 0-50 |  |  |  |  | 0-2 | 3-50 |
| *Platycarya longipes* | 0-50 |  |  |  |  |  | 0-50 |
| *Polygala wattersii* | 0-50 |  |  |  |  | 0-1 | 2-50 |
| *Pterospermum heterophyllum* | 0-50 |  |  |  | 0-7 | 8-44 | 45-50 |
| *Pyracantha fortuneana* | 0-50 |  |  |  | 0-2 | 2-17 | 18-50 |
| *Quercus engleriana* | 0-50 |  |  |  |  | 0-2 | 3-50 |
| *Radermachera sinica* | 0-50 |  |  |  | 0-29 | 30-50 |  |
| *Rapanea kwangsiensis* | 0-50 |  |  |  |  | 0-46 | 47-50 |
| *Rapanea neriifolia* | 0-50 |  |  |  |  | 0-1 | 2-50 |
| *Rhamnus subapetala* | 0-50 |  |  |  |  | 0-50 |  |
| *Rhus chinensis* | 0-50 |  |  |  |  | 0-50 |  |
| *Rubovietnamia aristata* | 0-50 |  |  |  | 0-8 | 9-45 | 46-50 |
| *Sapium rotundifolium* | 0-50 |  |  |  |  | 0-17 | 18-50 |
| *Sinoadina racemosa* | 0-50 |  |  |  | 0-4 | 5-30 | 31-50 |
| *Sinosideroxylon pedunculatum* | 0-50 |  |  |  |  | 0-7 | 8-50 |
| *Sinosideroxylon wightianum* | 0-50 |  |  |  |  | 0-27 | 28-50 |
| *Sterculia euosma* | 0-50 |  |  |  |  | 0-14 | 15-50 |
| *Sterculia lanceolata* | 0-50 |  |  |  | 0-7 | 8-24 | 25-50 |
| *Sterculia nobilis* | 0-50 |  |  |  |  | 0-17 | 18-50 |
| *Swida parviflora* | 0-50 |  |  |  |  | 0-27 | 28-50 |
| *Syzygium rehderianum* | 0-50 |  |  |  |  | 0-50 |  |
| *Tarenna attenuata* | 0-45 | 46-50 |  |  |  | 0-45 | 46-50 |
| *Tarenna depauperata* | 0-50 |  |  |  |  | 0-26 | 27-50 |
| *Tirpitzia ovoidea* | 0-50 |  |  |  |  | 0-1 | 2-50 |
| *Tirpitzia sinensis* | 0-50 |  |  |  |  | 0-18 | 19-50 |
| *Toona sinensis* | 0-50 |  |  |  |  | 0-50 |  |
| *Toxicodendron succedaneum* | 0-46 | 47-50 |  |  |  | 0-50 |  |
| *Turpinia montana* | 0-50 |  |  |  |  | 0-43 | 44-50 |
| *Viburnum triplinerve* | 0-50 |  |  |  |  | 0-7 | 8-50 |
| *Vitex canescens* | 0-48 | 49-50 |  |  | 0-11 | 12-50 |  |
| *Xylosma controversum* | 0-50 |  |  |  | 0-9 | 10-50 |  |
| *Zanthoxylum armatum* | 0-50 |  |  |  | 0-4 | 5-50 |  |
| *Zenia insignis* | 0-50 |  |  |  |  | 0-50 |  |
| *Ziziphus incurva* | 0-50 |  |  |  |  | 0-50 |  |

Note: AG, RA, and RE denote aggregated, random, and regular, respectively.
